# Tailoring manganese oxide with atomic precision to increase surface site availability for oxygen reduction catalysis

**DOI:** 10.1038/s41467-018-06503-8

**Published:** 2018-10-02

**Authors:** C. John Eom, Ding-Yuan Kuo, Carolina Adamo, Eun Ju Moon, Steve J. May, Ethan J. Crumlin, Darrell G. Schlom, Jin Suntivich

**Affiliations:** 1 Department of Materials Science and Engineering, Cornell University, Ithaca, NY 14853 USA; 2 Department of Applied Physics, Stanford University, Stanford, CA 94305 USA; 3 Department of Materials Science and Engineering, Drexel University, Philadelphia, PA 19104 USA; 4 Advanced Light Source, Lawrence Berkeley National Laboratory, Berkeley, CA 94720 USA; 5 Kavli Institute at Cornell for Nanoscale Science, Cornell University, Ithaca, NY 14853 USA

## Abstract

Controlling the structure of catalysts at the atomic level provides an opportunity to establish detailed understanding of the catalytic form-to-function and realize new, non-equilibrium catalytic structures. Here, advanced thin-film deposition is used to control the atomic structure of La_2/3_Sr_1/3_MnO_3_, a well-known catalyst for the oxygen reduction reaction. The surface and sub-surface is customized, whereas the overall composition and *d*-electron configuration of the oxide is kept constant. Although the addition of SrMnO_3_ benefits the oxygen reduction reaction via electronic structure and conductivity improvements, SrMnO_3_ can react with ambient air to reduce the surface site availability. Placing SrMnO_3_ in the sub-surface underneath a LaMnO_3_ overlayer allows the catalyst to maintain the surface site availability while benefiting from improved electronic effects. The results show the promise of advanced thin-film deposition for realizing atomically precise catalysts, in which the surface and sub-surface structure and stoichiometry are tailored for functionality, over controlling only bulk compositions.

## Abstract (web summary)

Controlling structures at the atomic level provides an opportunity to design and understand catalysts. Here the authors use thin-film deposition to fabricate perovskite heterostructures in a non-equilibrium manner to assess the effects on electrocatalytic activity for oxygen reduction.

## Introduction

Understanding the structure–activity relationship of a catalyst at the atomic level has been a long-standing challenge in the pursuit of a designer catalyst^1–5^. Traditionally, researchers identify this relationship by varying the bulk structure and/or composition and examining the impact on the catalytic performance. Although these efforts have resulted in design principles and discoveries of new catalysts, options beyond bulk variable control have not been fully explored. Advances in non-platinum catalysts such as carbon-based and metal-oxide catalysts for alkaline conditions further accentuate the need for full exploration of catalyst design beyond bulk variable control^6,7^. An emerging concept is to independently tune the structure and composition in the surface and sub-surface layers, the layers most sensitive to surface reactions. Control of these parameters provide the allure of optimizing electronic structure and surface chemistry in separate but closely coupled atomic layers^8,9^. Independently controlling the surface and sub-surface structure and composition, however, requires the ability to prepare materials with atomic precisions in a non-equilibrium manner. This often presents a practical obstacle for materials scientists. The purpose of this article is to present a route to realize this concept via advanced thin-film deposition.

Studies of well-defined single crystals have shown that the sub-surface atoms play an important role on the catalytic performance just as the surface atoms^10–15^. Driven by these findings, many researchers have synthesized catalysts with core-shell structures to manipulate the performance of the catalyst by using heat treatment to induce surface segregation or via post-synthetic deposition^16–19^. These syntheses, however, do not afford the ability to control the surface and sub-surface structure and composition in a systematic fashion. As a result, it has not been straightforward to obtain the structure–activity relations from these experiments. The difficulty in controlling the surface and sub-surface structure and composition is particularly challenging for oxide catalysts used in alkaline fuel cells and metal-air batteries, where complex quaternary phase diagrams and high-temperature treatment used for preparations often push the catalyst to favor the equilibrium structure^20–26^.

Here, this difficulty is tackled through advanced thin-film deposition. Thin films of La_2/3_Sr_1/3_MnO_3_ are grown layer-by-layer by molecular beam epitaxy (MBE). The surface and sub-surface compositions of the films were varied by tuning the deposition sequence while maintaining the overall stoichiometry and identical electronic structure. Comparison of the catalytic activity of the films for oxygen reduction reaction (ORR) show that the placement of the SrMnO_3_ layer in the sub-surface shows greater activity than when SrMnO_3_ is placed on the surface. Ambient pressure X-ray photoelectron spectroscopy (APXPS) reveals that the SrMnO_3_ layer improves the electronic structure and conductivity for ORR, but simultaneously reacts with ambient air to reduce the number of active sites. Placing SrMnO_3_ in the sub-surface is the optimal choice for the film to maximally benefit from the former effect, whereas minimizing the latter.

## Results

### Film growth and structural characterization

**Fig. 1:**
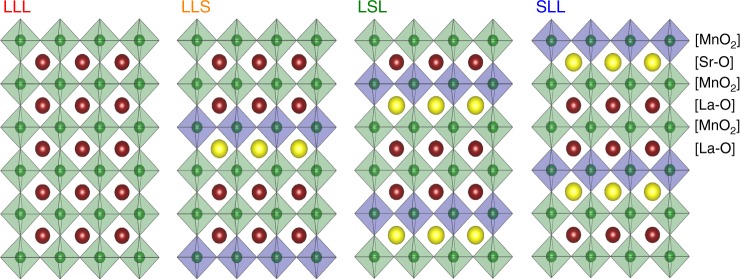
Schematic illustrations of the (LaMnO_3_)_2_/(SrMnO_3_) heterostructures in this work. The naming scheme refers to the order of the A-site metals beginning at the surface layer. LaMnO_3_-LaMnO_3_-LaMnO_3_ as “LLL”, LaMnO_3_-LaMnO_3_-SrMnO_3_ as “LLS”, LaMnO_3_-SrMnO_3_-LaMnO_3_ as “LSL”, and SrMnO_3_-LaMnO_3_-LaMnO_3_ as “SLL.” Blue manganite octahedra are associated with the Sr-O layer (yellow) and the green manganite octahedra are associated with the La-O layer (maroon)

**Fig. 2:**
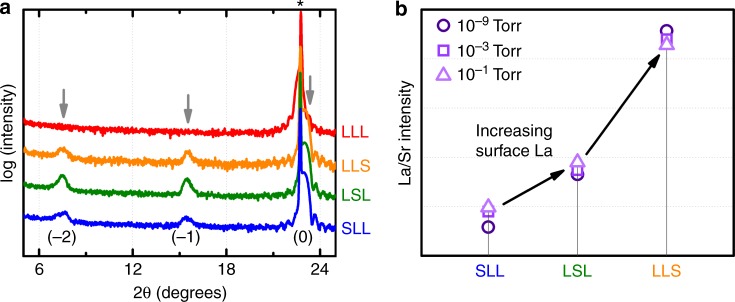
Structure and near-surface composition of (LaMnO_3_)_2_/(SrMnO_3_) heterostructures. **a** X-ray diffraction patterns of (LaMnO_3_)_2_/(SrMnO_3_) films. Peaks near 23° can be assigned to the (001) pseudo-cubic perovskite structure and the substrate peak (003 and *, respectively). The peaks near 14° and 7° are assigned to the satellite reflections of the superlattice (−1 and −2) from the cation ordering. **b** Ambient pressure X-ray photoelectron spectroscopy measurement of the La/Sr intensity ratio of the (LaMnO_3_)_2_/(SrMnO_3_) heterostructures at different oxygen pressures. LaMnO_3_-LaMnO_3_-LaMnO_3_ is referred to as “LLL”, LaMnO_3_-LaMnO_3_-SrMnO_3_ as “LLS”, LaMnO_3_-SrMnO_3_-LaMnO_3_ as “LSL”, and SrMnO_3_-LaMnO_3_-LaMnO_3_ as “SLL”

In all, 20-nm thick La_2/3_Sr_1/3_MnO_3_ heterostructures were grown by MBE on TiO_2_-terminated (001) SrTiO_3_ single-crystal substrates^27^. (001) was chosen as the low-energy termination of the perovskite structure which allows for a layer-by-layer growth. The structural integrity of the heterostructures were characterized using four-circle X-ray diffraction (XRD), angle-resolved photoemission spectroscopy (ARPES), and transport measurements^28,29^. These results also show that thin films studied in this article, thin films of low repeat units (*n* = 1 and 2), the LaMnO_3_ and SrMnO_3_ layers are commensurate to the substrate and the effects of interlayer strain are very small. For comparison, we study LaMnO_3_ (LLL) and La_2/3_Sr_1/3_MnO_3_ (where La and Sr form a solid solution) films of the same thickness. For (LaMnO_3_)_2_/(SrMnO_3_), we permute the ordering between LaMnO_3_ and SrMnO_3_ to control the SrMnO_3_ placement at either the surface, sub-surface, and sub-sub-surface. Films are referred to be based on their repeat units: SrMnO_3_-LaMnO_3_-LaMnO_3_ as “SLL”, LaMnO_3_-SrMnO_3_-LaMnO_3_ as “LSL”, and LaMnO_3_-LaMnO_3_-SrMnO_3_ as “LLS” (Fig. 1). In this nomenclature, the first letter of the name represents the layer that is closest to the surface. We follow the same naming convention for (LaMnO_3_)_4_/(SrMnO_3_)_2_, where we prepare LLSSLL and SSLLLL as our model (LaMnO_3_)_4_/(SrMnO_3_)_2_ heterostructures (Supplementary Figure 1). X-ray analysis shows reflections from both the perovskite structure and the superlattice (Fig. 2a and Supplementary Figure 2).

To verify the SrMnO_3_ placement, we measure the intensity ratios between the raw La 4*d* and Sr 3*d* peaks using APXPS. For excitation energies that correspond to probing depths of around one functional unit (~1.1 nm) from the surface (photon energies of 340 eV for La 4*d* and 385 eV for Sr 3*d*), the APXPS results show that LLS has the highest La/Sr intensity ratio near the surface, followed by LSL, and then SLL (Fig. 2b). These compositional ratios agree with our intended SrMnO_3_ placement and are unchanged under different *p*O_2_ (Fig. 2b). The consistent compositional ratios support our naming nomenclature and our assignment of any observed difference in the ORR activity to the SrMnO_3_ placement within the heterostructures.

### ORR activity comparison

**Fig. 3:**
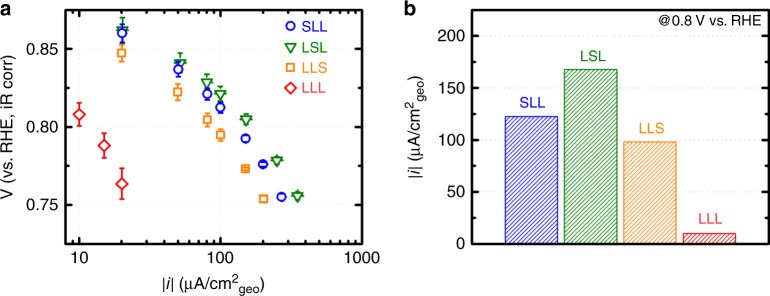
Oxygen reduction reaction activities of (LaMnO_3_)_2_/(SrMnO_3_). **a** Tafel plot of the oxygen reduction reaction (ORR) of (LaMnO_3_)_2_/(SrMnO_3_) in 0.1 M KOH. Error bars represent standard deviations of three independent measurements using three pieces of films deposited under the same condition. **b** A comparison of the ORR activities of (LaMnO_3_)_2_/(SrMnO_3_) at 0.8 V vs. reversible hydrogen electrode (RHE). LaMnO_3_-LaMnO_3_-LaMnO_3_ is referred to as “LLL”, LaMnO_3_-LaMnO_3_-SrMnO_3_ as “LLS”, LaMnO_3_-SrMnO_3_-LaMnO_3_ as “LSL”, and SrMnO_3_-LaMnO_3_-LaMnO_3_ as “SLL”

The surface and sub-surface placement plays an important role on the ORR. Figure 3a shows the ORR activity on (LaMnO_3_)_2_/(SrMnO_3_) heterostructures in a Tafel plot. All (LaMnO_3_)_2_/(SrMnO_3_) heterostructures share the same Tafel slope, suggesting a similar reaction pathway (for the studied composition, the overall reaction is O_2_ + 2H_2_O + 4e^–^ → 4OH^–24^). As our analysis focuses on the current range that is much smaller than the mass transport limit^30^, we did not apply any diffusion correction. For a visual comparison, Fig. 3b shows the ORR current at 0.8 V vs. reversible hydrogen electrode (RHE). LSL, where the Sr layer resides in the sub-surface layer, is the most active, followed by SLL, LLS, and then LLL. The low activity of LLS suggests that Sr most efficiently benefits ORR when it is placed within two-unit cells from the surface Mn (<1 nm). As Sr benefits ORR by affecting the *d-*electron configuration of Mn in the *e*_*g*_ symmetry^20^, the low activity of LLS indicates that electronic effect of Sr is localized to approximately two-unit cells. This hypothesis is consistent with the observation that the ORR kinetics on (LaMnO_3_)_2_/(SrMnO_3_) is more active than (LaMnO_3_)_4_/(SrMnO_3_)_2_; both LLSSLL and SSLLLL (Supplementary Figures 3, 4) reveal that larger superlattice spacing impedes Sr from benefiting the topmost Mn layer for ORR. This interpretation of our results agrees with previous ARPES experiments and first-principle calculations. ARPES showed reduced electronic hopping integrals when the spacing between the LaMnO_3_ and SrMnO_3_ layers increases^28^. First-principle calculations showed that the charge transfer effects of the (LaMnO_3_)_2_/(SrMnO_3_) interfaces are confined to ~2-unit cells^31,32^. Comparing the electrochemical behavior of (LaMnO_3_)_2n_/(SrMnO_3_)n (*n* = 1, 2) to that of a random alloy (‘Alloy’) further reveals the effects of sub-surface engineering (Supplementary Figure 5). The Alloy film, which should structurally represent a random mixture of the three (LaMnO_3_)_2_/(SrMnO_3_) films, performs close to the average of the three films, markedly poorer than LSL (Supplementary Figure 5). Both our results and those in the literature from the electronic structure community confirm a long-held intuition: sub-surface chemistry manipulation is the most direct way to influence the activity of the topmost catalytic layer, and that the effect gradually decreases as the manipulation occurs on a layer further away from the surface.

Having concluded the positive role of the near-surface La-Sr interface, we can now discuss why ORR is more active when Sr is placed in the sub-surface (LSL) compared with when Sr is placed on the surface (SLL). Stoerzinger et al. have shown that the charge transfer capability in La_1-x_Sr_x_MnO_3_ (measured using the [Fe(CN)_6_]^3−/4−^ outer sphere redox reaction) depends on x, the Sr content. Their results suggested that in addition to tuning the *e*_*g*_ occupation, the Sr addition also benefits the ORR activity of LaMnO_3_ by providing carriers to increase conductivity^30^. Interestingly, we observe no difference in the charge transfer capabilities between LLS, LSL, and SLL, for the same [Fe(CN)_6_]^3−/4−^ outer sphere redox reaction (Supplementary Figure 6). Therefore, the availability of transferrable electrons does not explain the ORR difference between SLL, LSL, and LLS (Fig. 2). The observation that (LaMnO_3_)_2_/(SrMnO_3_) is not limited by the availability of transferrable electrons is consistent with the transport measurements, which showed that (LaMnO_3_)_2_/(SrMnO_3_) is metallic^29,33,34^.

### APXPS analysis

With conductivity ruled out, we study the surface chemistry and electronic structure using APXPS. Supplementary Figure 7 shows the valence band spectra at low-energy (340 eV, inelastic mean free path, IMFP, ~0.6 nm) and high-energy (780 eV, IMFP, ~1.3 nm) excitations in both vacuum (*p*O_2_ < 10^−9^ Torr) and near-ambient pressure (*p*O_2_ ~ 10^−1^ Torr). The valence spectra of La_1-x_Sr_x_MnO_3_ is principally assigned to a combination of the O 2*p* (6 eV) and Mn 3*d* states (2 eV)^35–38^. As we move from deeper probing depth (~1.3 nm) to shallower probing depth (~0.6 nm), the valence spectra undergo a spectral weight transfer from the high-energy edge to about 2 eV from the Fermi level. This change is attributed to the surface localization, which reduces the bandwidth and produces a localized, narrow Mn 3*d* state at the surface. These experiments have two implications. First, the use of a molecular orbital model is a reasonable approximation for the oxide surfaces given the localized nature of the Mn *d*-state. Second, even with synchrotron APXPS, the difference in the valence band spectra between the superlattices are not resolvable. We have therefore focused our analysis on the core spectra, which have superior signal-to-noise ratios.

**Fig. 4:**
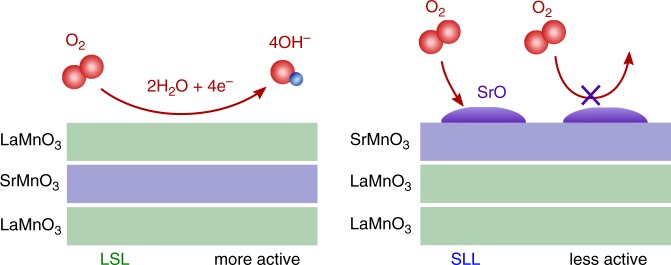
Schematic of proposed mechanism of surface oxygen interaction. LaMnO_3_-SrMnO_3_-LaMnO_3_ (LSL) interacts with oxygen by adsorbing oxygen atoms without significantly changing surface structure. SrMnO_3_-LaMnO_3_-LaMnO_3_ (SLL) interaction with oxygen involves migration of the A-site Sr atoms to the surface to form Sr-O like bonds

**Fig. 5:**
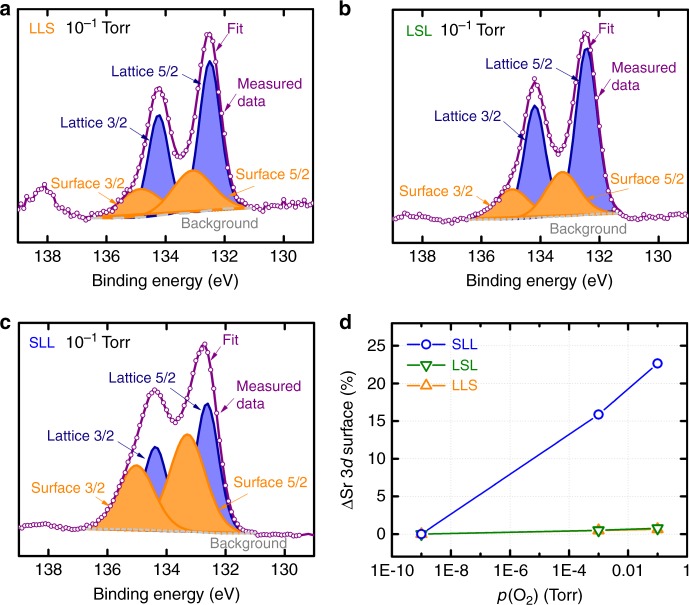
Sr 3*d* ambient pressure X-ray photoelectron spectra and compositional fits. Sr 3*d* ambient pressure x-ray photoelectron spectroscopy (APXPS) background-subtracted spectra and compositional fits for **a** LLS, **b** LSL, and **c** SLL at *p*(O_2_) at 10^−1^ Torr. The orange doublet corresponds to the surface Sr component, which we attribute to the Sr-oxide, whereas the blue doublet corresponds to the lattice Sr component within the bulk film. **d** The change in surface Sr component concentration (*p*(O_2_) – 10^−9^ Torr) grows systematically with *p*(O_2_) for SLL, whereas remain constant for LSL and LLS, suggesting the reaction of the surface Sr species in SLL upon contacting atmospheric oxygen. LaMnO_3_-LaMnO_3_-LaMnO_3_ is referred to as “LLL”, LaMnO_3_-LaMnO_3_-SrMnO_3_ as “LLS”, LaMnO_3_-SrMnO_3_-LaMnO_3_ as “LSL”, and SrMnO_3_-LaMnO_3_-LaMnO_3_ as “SLL”

The core spectra provide insight into the surface chemistry difference between LSL and SLL. Background-subtracted spectra show that La 4*d* peaks are insensitive of the SrMnO_3_ placement and the *p*O_2_ level (Supplementary Figure 8). This observation indicates that La is mostly a spectator and does not interact with atmospheric oxygen. We similarly observe that Sr 3*d* peaks in LSL and LLS do not change with *p*O_2_, which is consistent with the idea that the sub-surface and sub-sub-surface Sr cannot “see” atmospheric oxygen (Supplementary Figure 9). However, for SLL, APXPS reveals that Sr can react with oxygen to form Sr-oxide-like species (Fig. 4, Supplementary Figure 9). This surface Sr component (~135 eV) grows systematically with *p*O_2_ (Fig. 5, see Supplementary Figure 9 and Supplementary Table 1 for spectra analysis). Surface segregation of Sr-oxide like species have been observed for La_1-x_Sr_x_MnO_3_ powders, pellets, and films^39–41^. We similarly believe that the top SrO layer within the superlattice could segregate past the top MnO_2_ layer to form Sr-oxide species on the surface of SLL. These observations suggest that the formation of the surface Sr-oxide could block the oxygen access to the surface Mn once the catalyst is exposed to atmospheric oxygen during the ORR. In this picture, LSL is more active than SLL because SrMnO_3_ resides in the sub-surface layer. Even though their surfaces nominally share the same local electronic structure, LSL surface is more accessible due to the absence of blocking Sr oxides.

Surface site availability for oxygen adsorption plays a critical role in ORR on Pt. Stamenkovic et al. have suggested that the surface site availability enhances the ORR activity on Pt_3_Ni(111)^10^. We believe that a similar phenomenon occurs for the LSL heterostructure. The presence of Sr in LaMnO_3_ (i.e., La_2/3_Sr_1/3_MnO_3_), although beneficial by modifying the *e*_g_ occupation of the Mn to increase the ORR activity, can simultaneously reduce the surface site availability by forming a surface Sr-oxide group that restricts the oxygen access to the active Mn sites. Strmcnik et al. have previously suggested that the surface oxide species could reduce the surface site availability by interacting with the ions in the electrochemical double layer^42^. This scenario is also possible in our superlattices.

Our result shows the double-edged effect of Sr in La_2/3_Sr_1/3_MnO_3_, where Sr can promote ORR by modifying the Mn electronic structure, but suppress the surface site availability when placed too close to the surface. The double-edged effect may explain why increasing the Mn valence state by oxygen overstoichiometry (e.g., LaMnO_3+ δ_) results in a more active catalyst than by aliovalent substitutions (e.g., La_1-x_Ca_x_MnO_3_), despite both leading to Mn catalysts with nearly the same Mn oxidation state and crystal structure^20^. We emphasize, however, that this is unlikely the only effect. As recently highlighted, Mn perovskite oxides are more active when synthesized using a co-precipitation method^20,43,44^. Defects and surface segregation likely play a critical role also. Finally, we note that we were unable to resolve major differences between the superlattices in either the valence band spectra, O 1*s* (K-edge), or Mn 2*p* (L-edge) X-ray absorption (Supplementary Figures 10, 11), which likely points to the need for higher resolution, higher efficiency X-ray techniques, especially new detector technologies that can resolve local electronic structure in the near-surface layer. Previous X-ray studies on La_1-*x*_Sr_*x*_MnO_3_ have shown only subtle changes in valence band for 0.3 ≤ *x* ≤ 0.6 despite observable differences in electronic properties^35,45^. Developing finer resolution spectroscopy methods is an essential future step to reveal the nature of surface bonding in transition-metal oxides designed with customized surface and sub-surface.

## Discussion

Our investigation of the surface and sub-surface customizations of La_2/3_Sr_1/3_MnO_3_ reveals that ORR is most active when SrMnO_3_ resides in the sub-surface vs. when it resides in the surface, the sub-sub-surface, or randomly distributed. APXPS reveals that this observation stems from the double-edged effect of Sr, which benefits ORR by modifying the electronic structure of the Mn sites, but can also form non-catalytic Sr oxides that reduce the surface site availability. Analysis of the valence band and elemental peak spectra in the presence of oxygen reveals that Sr modifies the electronic structure but also reacts to form a detrimental oxide species when present in the surface layer. Our finding demonstrates the Goldilocks effect, where moving the Sr closer to the surface allows the surface Mn atoms to more efficiently catalyze ORR, but Sr at the surface poisons the catalyst and reduces functionality. As a result, the catalyst is most active when the surface and sub-surface layer are LaMnO_3_ and SrMnO_3_, respectively. Most importantly, our work points to an opportunity to design an ORR catalyst by customizing surface and sub-surface stoichiometry, orientation, and structure. The ability to control both parameters independently and rationally with atomic-layer precision can lead to the discovery of new catalysts with functionalities superior to those accessed through traditional bulk-composition control.

## Methods

### MBE

Epitaxial (LaMnO_3_)_2*n*_/(SrMnO_3_)_*n*_ (*n* = 1, 2) superlattices were deposited on (001)-SrTiO_3_ single crystals using MBE. LaMnO_3_ (LLL), La_2/3_Sr_1/3_MnO_3_ and five superlattices (LLS, LSL, SLL, LLSSLL, and SSLLLL) were prepared using a layer-by-layer deposition. In all synthesized heterostructures, the first letter represents the surface layer. All films were terminated with the Mn layer and XRD confirms the pseudo-cubic perovskite structure.

### APXPS

To establish the connection between the surface and sub-surface customizations and electronic structure, we use APXPS^46^ to monitor the surface chemistry of the heterostructures. APXPS measurements were collected at Beamline 9.3.2 at the Advanced Light Source (ALS) of the Lawrence Berkeley National Laboratory (LBNL). After loading, all samples were heated to and held at 200 °C at 1 mTorr *p*O_2_ for 30 min to remove surface adsorbed organics, which were monitored via the C1s spectra. We tune to the excitation energy such that the kinetic energy of the escaping electron is uniformly 250 eV for all elemental peaks. This kinetic energy corresponds to a probing depth of around one functional unit (~1.1 nm). The following elemental peaks were measured (at the specified excitation energies): O 1*s* (780 eV), Sr 3*d* (385 eV), and La 4*d* (340 eV). During the core peak measurements, we simultaneously collect the valence spectra. La 4*d* XPS peaks were calibrated to the leading edge of the La 4*d* peaks to be at 100 eV as La peaks are qualitatively unaffected by changes in *p*O_2_ (Fig. S1). Other peaks were calibrated with respect to the La 4*d* peaks of the same sample and pressure by aligning the O 2*p* edge of the valence band spectra (7–8 eV) to the calibrated valence band spectra of the La 4*d* peaks with an error of ± 0.1 eV. We use a Shirley background correction. We also collected Mn L-edge and O K-edge X-ray absorption spectra in a partial-electron-yield mode in the same conditions as APXPS. All spectra were normalized to the background absorption below and above the absorption edge.

### Electrochemical characterizations

Electrochemical measurements were conducted in a standard three-electrode cell (Pine) using a potentiostat (Bio-Logic). The reference electrode was a Ag/AgCl redox couple in a saturated KCl solution, calibrated to the H_2_/H^+^ redox. The ohmic resistance correction used the high-frequency real-axis intercept of the impedance measurement. Front contacts were made using gallium-indium eutectic (Sigma-Aldrich, 99.99%) and silver paint (Ted Pella, Leitsilber 200) for electrical contact. The contact and the sides and back of the substrate were all covered with epoxy (Omegabond 101) to ensure electrochemical insulation of these regions^30^. All samples were front contacted to avoid passing the current through the film-substrate interface. The ORR measurements were conducted in O_2_-saturated (Airgas, ultrahigh-grade purity) 0.1 M KOH, prepared from Milli-Q water (18.2 Ω∙cm, Millipore) with KOH pellets (Sigma-Aldrich, 99.99%) at a rate of 10 mV/s. The ORR curves were obtained by subtracting the ORR current measured in O_2_ vs. background current measured in Ar (Airgas, ultrahigh-grade purity) in 0.1 M KOH. The ORR activity is from the third cycle, where the subsequent scan did not vary by >5% of the previous scan. The error bars represent the standard deviations for at least three independent measurements. The [Fe(CN)_6_]^3−/4−^ measurements were conducted in Ar-saturated 0.1 M KOH solution with 5 mM K_4_Fe(CN)_6_∙3H_2_O (Alfa Aesar, 98.5–102.0%) and K_3_Fe(CN)_6_ (Alfa Aesar, 99%) at a rate of 10 mV/s.

## Electronic supplementary material

Supplementary Information

## Data Availability

The data that support the findings of this study are available from the authors on reasonable request.
