# Supplementary material for: Tailoring manganese oxide with atomic precision to increase surface site availability for oxygen reduction catalysis
Source: Nat Commun. 2018 Oct 2;9:4034. doi: 10.1038/s41467-018-06503-8 (PMC6168596; doi:10.1038/s41467-018-06503-8)
Supplement: Supplementary file 1 — Supplementary Information [file 41467_2018_6503_MOESM1_ESM.pdf]

Supplementary Information (SI)

Tailoring Manganese-Oxide with Atomic Precision to Increase Surface Site Availability for Oxygen Reduction Catalysis

C. John Eom[a], Ding-Yuan Kuo[a], Carolina Adamo[b], Eun Ju Moon[c], Steve J. May[c], Ethan J. Crumlin[d], Darrell G. Schlom[a,e], Jin Suntvich[a,e,*]

[a] *Department of Materials Science and Engineering, Cornell University, Ithaca, New York 14850, USA;*
[b] *Department of Applied Physics, Stanford University, Palo Alto, CA 94306, USA;*
[c] *Department of Materials Science and Engineering, Drexel University, Philadelphia, PA 19104, USA;*
[d] *Advanced Light Source, Lawrence Berkeley National Laboratory, Berkeley, California 94720, USA;*
[e] *Kavli Institute at Cornell for Nanoscale Science, Cornell University, Ithaca, New York 14853, USA*
**Correspondence and requests for materials should be addressed to J.S. (email: jsuntivich@cornell.edu)*

Content

Structural characterization of the $(LMO)_4/(SMO)_2$ heterostructures (Supplementary Figures 1-2) 2

Electrochemical characterization of $(LMO)_2/(SMO)$, $(LMO)_4/(SMO)_2$, and 'Alloy' (Supplementary Figures 3-4) 3

Electrochemical characterization of (LMO)2/(SMO) and 'Alloy' (Supplementary Figure 5) 4

Outer-sphere-redox reaction of $(LMO)_2/(SMO)$ heterostructures (Supplementary Figure 6) 4-5

APXPS valence spectra of $(LMO)_2/(SMO)$ heterostructures (Supplementary Figure 7) 5-6

APXPS La *4d* spectra of $(LMO)_2/(SMO)$ heterostructures (Supplementary Figure 8) 6-7

APXPS Sr *3d* spectra of $(LMO)_2/(SMO)$ heterostructures (Supplementary Figure 9) 7

O K-edge spectra of $(LMO)_2/(SMO)$ heterostructures (Supplementary Figure 10) 8

Mn L-edge spectra of $(LMO)_2/(SMO)$ heterostructures (Supplementary Figure 11) 9

Fit parameters for the Sr *3d* peak analysis of $(LMO)_2/(SMO)$ (Supplementary Table 1) 10

Supplementary References 11

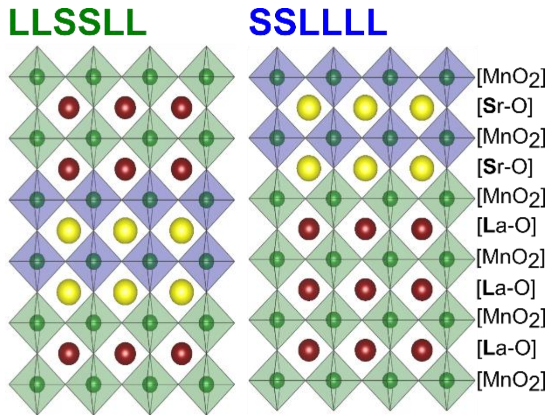

Supplementary Figure 1 Schematic illustrations of the $(LaMnO_3)_4/(SrMnO_3)_2$ heterostructures Naming scheme refers to the order of the A-site metals beginning at the surface layer. $LaMnO_3$-$LaMnO_3$-$SrMnO_3$-$SrMnO_3$-$LaMnO_3$-$LaMnO_3$ as "LLSSLLL" and $SrMnO_3$-$SrMnO_3$-$LaMnO_3$-$LaMnO_3$-$LaMnO_3$-$LaMnO_3$ as "SSLLLL."

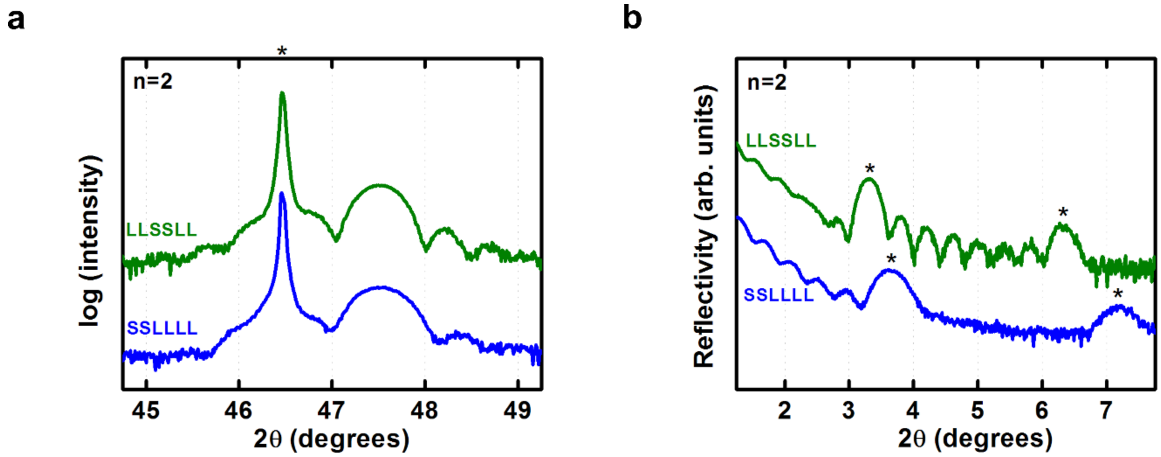

Supplementary Figure 2 (a) X-ray diffraction data of the (002) peak from the $(LaMnO_3)_4/(SrMnO_3)_2$ films Substrate $SrTiO_3$ peaks are denoted with an asterisk (*). (b) X-ray reflectivity data from the same superlattices; the peaks denoted with an asterisk (*) are from the Sr/La cation ordering in the superlattice.

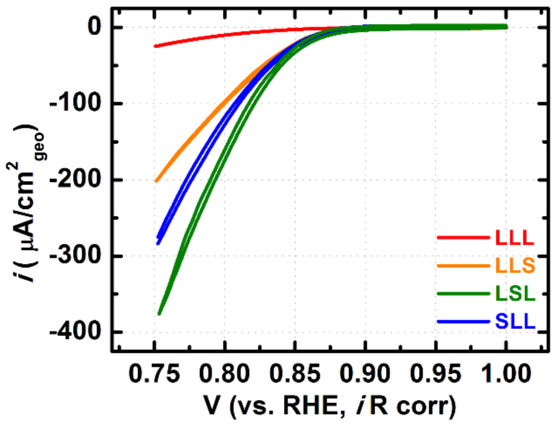

Supplementary Figure 3 ORR on $(LaMnO_3)_2/(SrMnO_3)$ heterostructures We use cyclic voltammetry at 10 mV/s rate in O_2-saturated 0.1 M KOH to measure the ORR current. LSL is the most ORR active, followed by SLL, LLS, and then LLL.

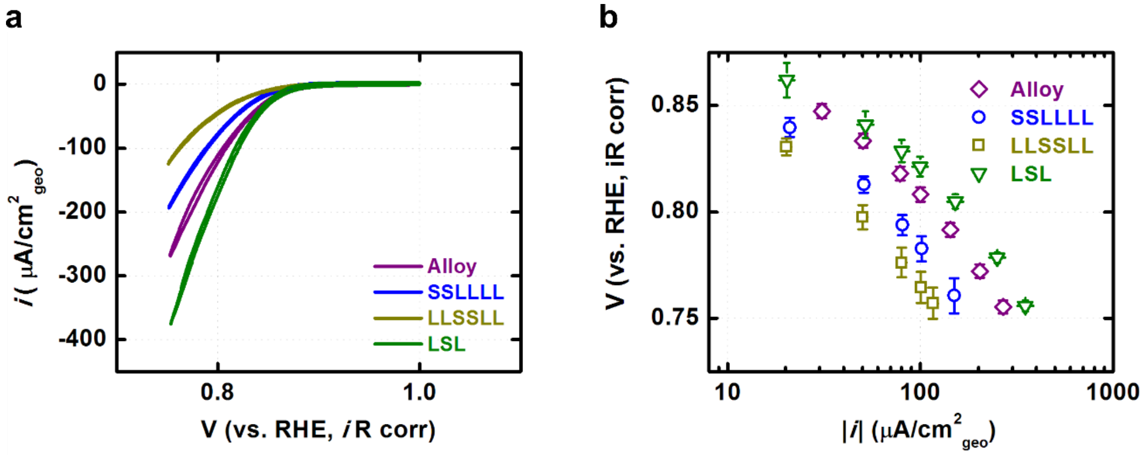

Supplementary Figure 4 ORR results of the $(LaMnO_3)_4/(SrMnO_3)_2$ heterostructures compared with La and Sr in a solid solution ('alloy'), and LSL. (a) Cyclic voltammetry of $(LaMnO_3)_4/(SrMnO_3)_2$ in O_2-saturated 0.1 M KOH. (b) Tafel plot of $(LaMnO_3)_4/(SrMnO_3)_2$ obtaining from the cyclic voltammetry results in (a), after a capacitance correction. Error bars represent the standard deviation of three measurements from three pieces of films deposited at the same condition.

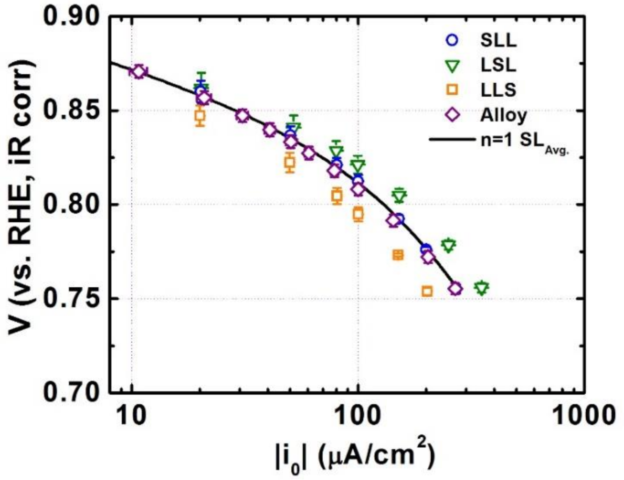

Supplementary Figure 5 ORR results of $(LaMnO_3)_2/(SrMnO_3)$ heterostructures compared with La and Sr in a solid solution ('alloy'), and average of the three $(LaMnO_3)_2/(SrMnO_3)$ heterostructures. The average is displayed for comparison with the alloy activity, not to claim causation.

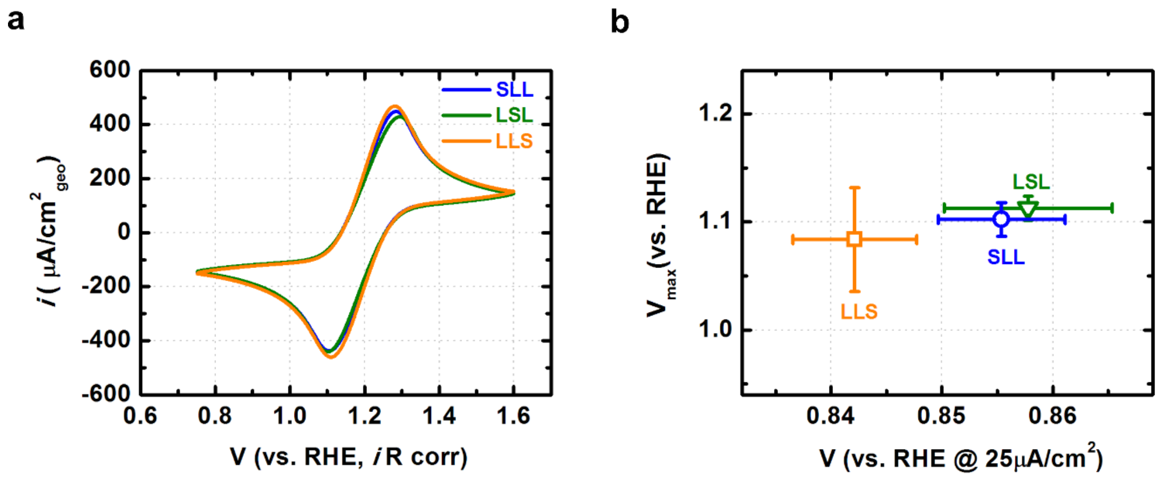

Supplementary Figure 6 Outer-sphere-redox $[Fe(CN)_6]^{3-/4-}$ reaction of the $(LaMnO_3)_2/(SrMnO_3)$ heterostructures. (a) Cyclic voltammetry of the outer-sphere-redox $[Fe(CN)_6]^{3-/4-}$ reaction on $(LaMnO_3)_2/(SrMnO_3)$. (b) A comparison between the peak current positions of the outer-sphere $[Fe(CN)_6]^{3-/4-}$ reaction and the ORR activity (defined to be the

potential, where the ORR current density is 25 μA/cm^2) on the $(LaMnO_3)_2/(SrMnO_3)$ heterostructures. We do not observe significant difference in outer-sphere-redox $[Fe(CN)_6]^{3-/4-}$ reaction between the $(LaMnO_3)_2/(SrMnO_3)$ heterostructures. Therefore, we believe that the enhancement mechanism in LSL is different from the charge-transfer mechanism proposed to explain different activities in $La_{1-x}Sr_xMnO_3$ with changing x. Horizontal error bars represent the standard deviation of three measurements from three pieces. Vertical error bars are calculated from the peak width at 90% from maximum current potential.

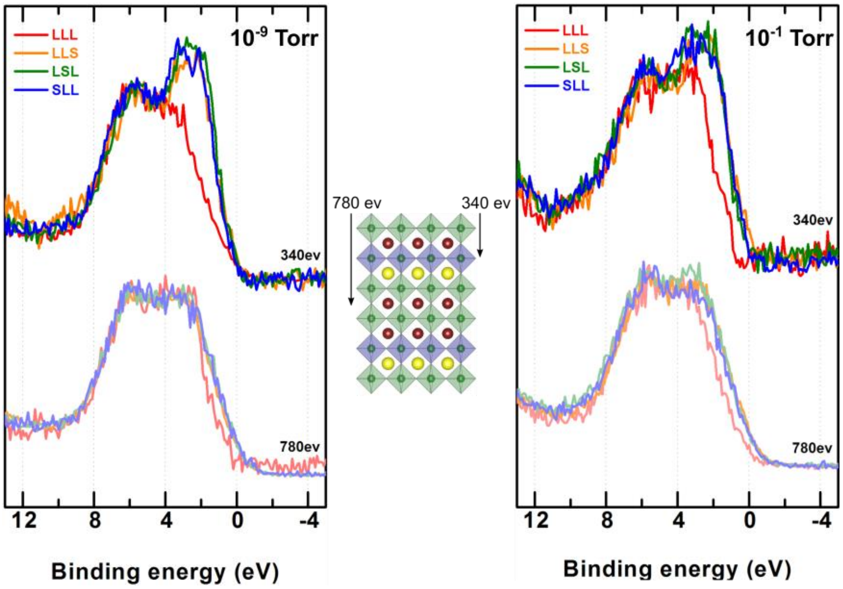

Supplementary Figure 7 Valence spectra of $(LaMnO_3)_2/(SrMnO_3)$ at different excitation energies and $p(O_2)$ values. In going from lower to higher excitation energy (increasing the probing depth), we observe a spectral-weight transfer at ~2 eV below the Fermi level. We attribute this observation to the surface Mn 3*d* localization. We, however, did not observe major difference in the valence spectra between different heterostructures; hence we propose that their surface – oxygen interactions are identical. The valence spectra are presented by aligning all spectra's band edges at 7-8 eV. At 340 eV, the inelastic mean free path (IMFP) is ~ 6 Å and at

780 eV, the IMFP is ~ 13 Å. The IMFPs were calculated based on the ratios of raw intensities of the La and Sr core peaks normalized to their respective photoemission cross sections as reported by Yeh and Lindau[1]. The ratio of the intensities against the expected depth of the Sr layer from the surface (~0.39 nm per layer) were fit to an exponential function. The inverse of the exponential constant was used as the IMFP at 250 eV (approximately 1.1 nm). Using the linearized part of the *universal curve*, the respective IMFPs for the X-ray energies were calculated.

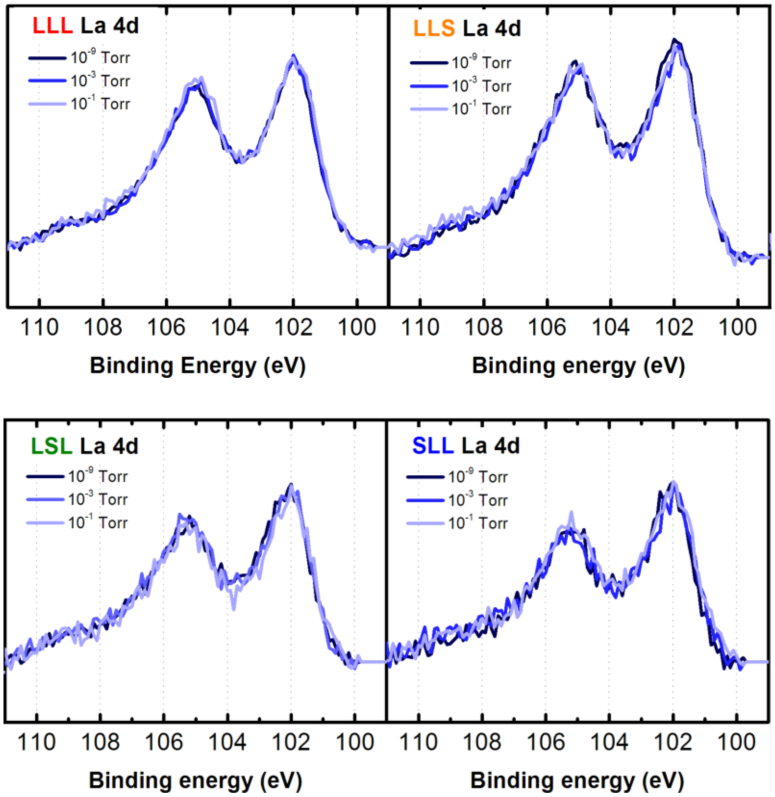

Supplementary Figure 8 APXPS spectra of the La 4*d* core peaks In all samples tested, background-corrected La 4*d* peaks show negligible $p(O_2)$ dependence, indicating that La does not interact with atmospheric oxygen. Naming scheme refers to the order of the A-site metals

---

beginning at the surface layer. $LaMnO_3$-$LaMnO_3$-$LaMnO_3$ as "LLL", $LaMnO_3$-$LaMnO_3$-$SrMnO_3$ as "LLS", $LaMnO_3$-$SrMnO_3$-$LaMnO_3$ as "LSL", and $SrMnO_3$-$LaMnO_3$-$LaMnO_3$ as "SLL."

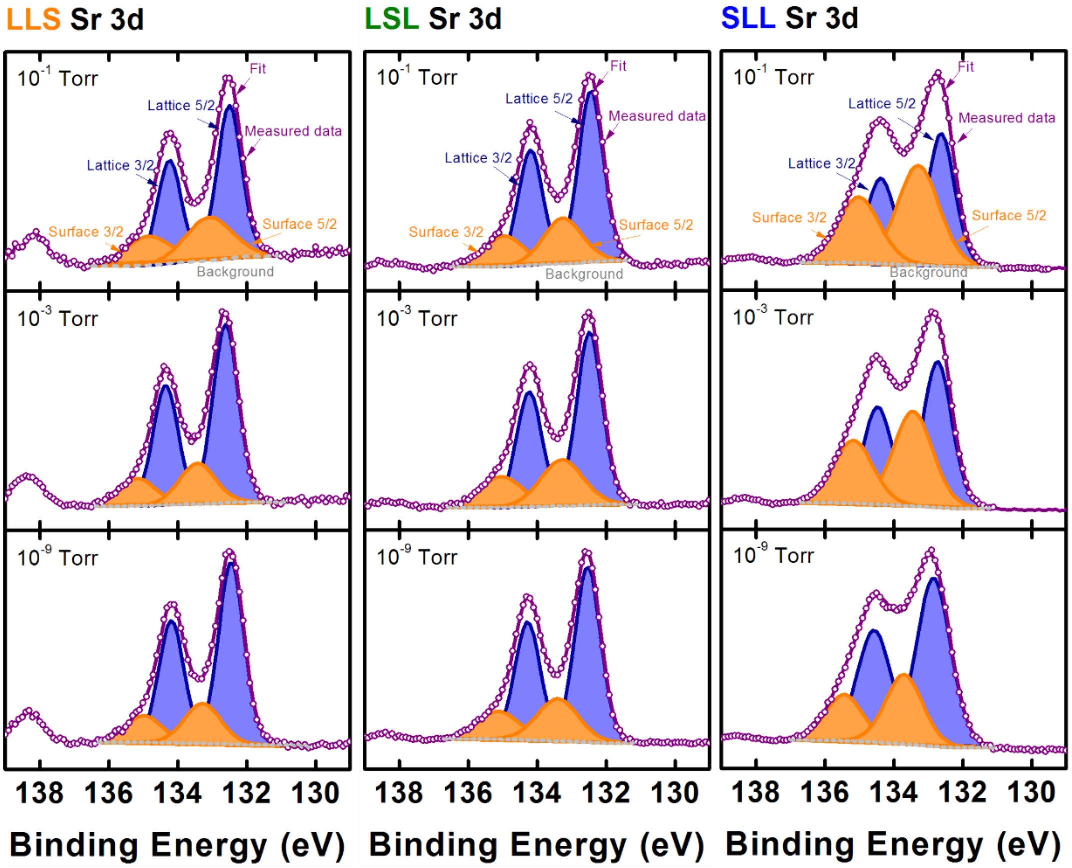

Supplementary Figure 9 APXPS spectra of the Sr 3*d* peaks and their compositional fits in $(LaMnO_3)_2/(SrMnO_3)$ heterostructures. For LLS and LSL, the surface (orange) and the bulk (blue) components do not change with $p(O_2)$. This observation stands in contrast to SLL, which shows increasing surface Sr species with increasing $p(O_2)$. Details of the fitting parameters for each peak are presented below in Table S1.

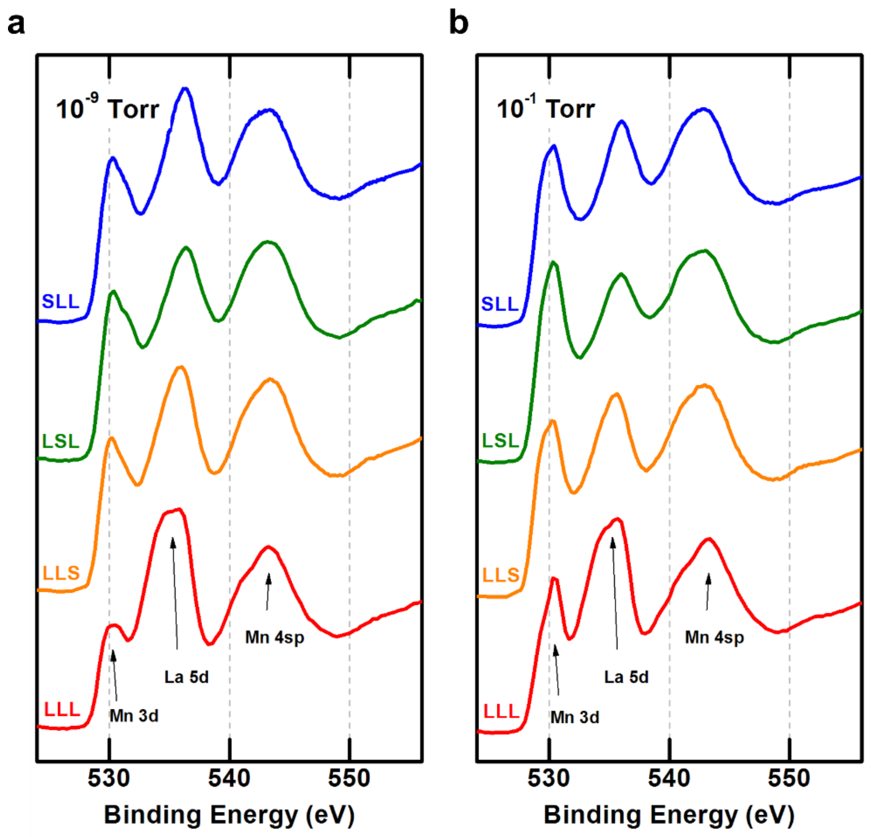

Supplementary Figure 10 O K-edge X-ray Absorption Spectroscopy of the $(LaMnO_3)_2/(SrMnO_3)$ heterostructures and $La_{0.67}Sr_{0.33}MnO_3$ alloy. (a) O K-edge X-ray absorption spectra at 10^{-9} Torr $p(O_2)$ and (b) at 10^{-1} Torr $p(O_2)$. O K-edge X-ray absorption spectra were collected in a partial-electron-yield mode in the same conditions as APXPS.

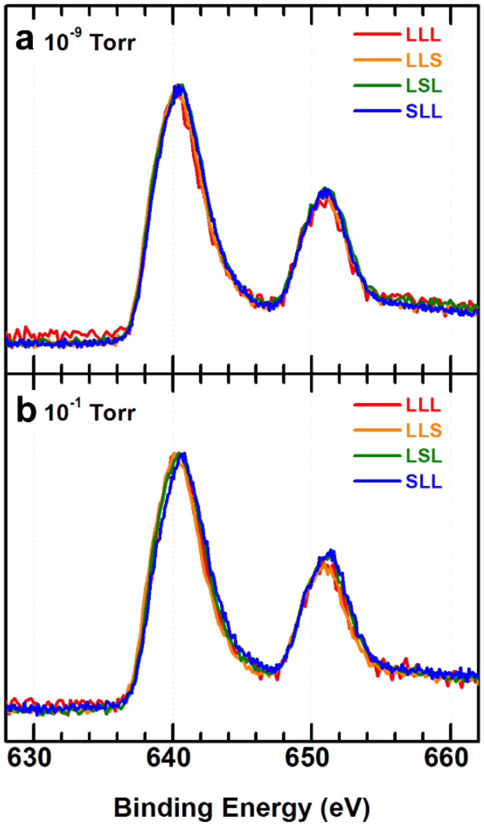

Supplementary Figure 11 Mn L-edge X-ray Absorption Spectroscopy of the $(LaMnO_3)_2/(SrMnO_3)$ heterostructures and $La_{0.67}Sr_{0.33}MnO_3$ alloy. (a) Mn L-edge X-ray absorption spectra at 10^{-9} Torr $p(O_2)$ and (b) at 10^{-1} Torr $p(O_2)$. We observe that the Mn L-edge is similar for all $(LMO)_2/(SMO)$ samples across $p(O_2)$ region tested. Mn L-edge X-ray absorption spectra were collected in a partial-electron-yield mode in the same conditions as APXPS.

Band Name	Position (eV)	FWHM (eV)	Area
LLS Sr 3d L 5/2 10^{-9} Torr	132.5	0.9	266371
LLS Sr 3d L 3/2 10^{-9} Torr	134.2	0.9	177580
LLS Sr 3d S 5/2 10^{-9} Torr	133.3	1.2	83262
LLS Sr 3d S 3/2 10^{-9} Torr	135.0	1.2	55508
LSL Sr 3d L 5/2 10^{-9} Torr	132.9	0.9	471838
LSL Sr 3d L 3/2 10^{-9} Torr	134.7	0.9	314559
LSL Sr 3d S 5/2 10^{-9} Torr	133.8	1.4	179982
LSL Sr 3d S 3/2 10^{-9} Torr	135.5	1.4	119994
SLL Sr 3d L 5/2 10^{-9} Torr	132.9	1.2	1266752
SLL Sr 3d L 3/2 10^{-9} Torr	134.6	1.2	844501
SLL Sr 3d S 5/2 10^{-9} Torr	133.8	1.1	414106
SLL Sr 3d S 3/2 10^{-9} Torr	135.5	1.1	276071
LLS Sr 3d L 5/2 10^{-6} Torr	132.6	0.9	225612
LLS Sr 3d L 3/2 10^{-6} Torr	134.4	0.9	150408
LLS Sr 3d S 5/2 10^{-6} Torr	133.4	1.2	70498
LLS Sr 3d S 3/2 10^{-6} Torr	135.1	1.2	46999
LSL Sr 3d L 5/2 10^{-6} Torr	132.9	0.9	377936
LSL Sr 3d L 3/2 10^{-6} Torr	134.6	0.9	251957
LSL Sr 3d S 5/2 10^{-6} Torr	133.7	1.4	162043
LSL Sr 3d S 3/2 10^{-6} Torr	133.5	1.4	108029
SLL Sr 3d L 5/2 10^{-6} Torr	132.7	1.0	775850
SLL Sr 3d L 3/2 10^{-6} Torr	134.5	1.0	517234
SLL Sr 3d S 5/2 10^{-6} Torr	133.4	1.3	685953
SLL Sr 3d S 3/2 10^{-6} Torr	135.2	1.3	457302
LLS Sr 3d L 5/2 10^{-1} Torr	132.5	0.9	180366
LLS Sr 3d L 3/2 10^{-1} Torr	134.3	0.9	120244
LLS Sr 3d S 5/2 10^{-1} Torr	133.4	1.1	45759
LLS Sr 3d S 3/2 10^{-1} Torr	135.1	1.1	30506
LSL Sr 3d L 5/2 10^{-1} Torr	132.8	0.9	302810
LSL Sr 3d L 3/2 10^{-1} Torr	134.6	0.9	201874
LSL Sr 3d S 5/2 10^{-1} Torr	133.5	1.5	157984
LSL Sr 3d S 3/2 10^{-1} Torr	135.3	1.5	105323
SLL Sr 3d L 5/2 10^{-1} Torr	132.6	0.9	575833
SLL Sr 3d L 3/2 10^{-1} Torr	134.4	1.0	383889
SLL Sr 3d S 5/2 10^{-1} Torr	133.3	1.4	670577
SLL Sr 3d S 3/2 10^{-1} Torr	135.0	1.4	442816

Supplementary Table 1 List of fit parameters for Sr *3d* peak analysis of $(LMO)_2/(SMO)$.

Supplementary References

1. Yeh, J. J. & Lindau, I. Atomic subshell photoionization cross sections and asymmetry parameters: $1 \leq Z \leq 103$. *At. Data Nucl. Data Tables* **32,** 1–155 (1985).
